# Concomitant Retroperitoneal and Subarachnoid Hemorrhage Due to Segmental Arterial Mediolysis: Case Report and Review of the Literature

**DOI:** 10.1007/s00062-017-0641-5

**Published:** 2017-11-03

**Authors:** V. Hellstern, M. Aguilar Pérez, P. Kohlhof-Meinecke, H. Bäzner, O. Ganslandt, H. Henkes

**Affiliations:** 1 Neuroradiologische Klinik, Klinikum Stuttgart, Kriegsbergstraße 60, 70174 Stuttgart, Germany; 2 Institut für Pathologie, Klinikum Stuttgart, Stuttgart, Germany; 3 Neurologische Klinik, Klinikum Stuttgart, Stuttgart, Germany; 4 Neurochirurgische Klinik, Klinikum Stuttgart, Stuttgart, Germany; 5 Medizinische Fakultät, Universität Duisburg-Essen, Essen, Germany

## Introduction

“Segmental mediolytic arteriopathy” or “segmental arterial mediolysis” (SAM) is an up to now idiopathic disorder of the visceral and intracranial arteries and is known as a cause of major abdominal, retroperitoneal and subarachnoid hemorrhage [1–3]. Recently, Pickup and Pollanen [4] suggested SAM to be a condition found in Ehlers–Danlos type IV. The affected arteries show a noninflammatory and nonatherosclerotic vacuolization and lysis of the tunica media, smooth muscle degeneration and serration of the lamina elastica interna. These alterations undermine the vessel wall stability. Spontaneous dissection and aneurysm formation, followed by aneurysm rupture may occur. SAM is the most likely diagnosis in the case of simultaneous abdominal or retroperitoneal and subarachnoid hemorrhage. We describe the case history of a patient with ruptured dissecting aneurysms of abdominal and intracranial arteries. The basilar artery aneurysm was treated by endovascular flow diversion.

## Case Report

**Fig. 1:**
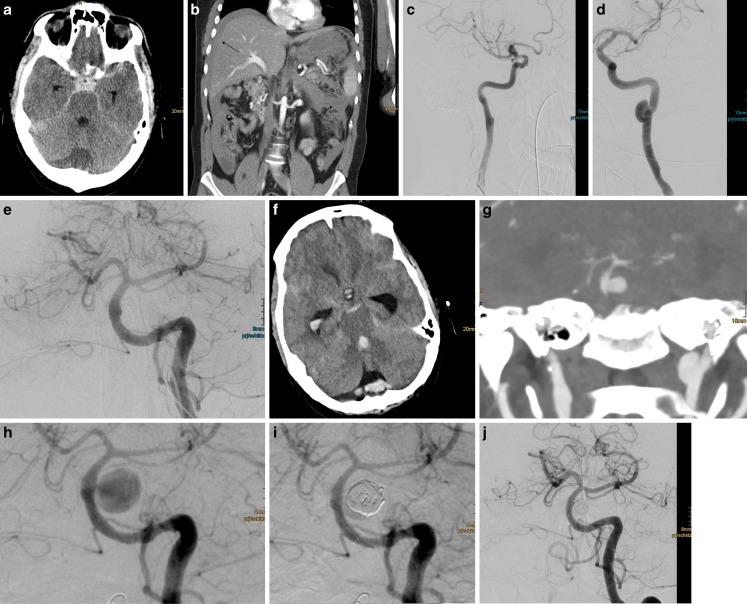
CT and DSA findings in a case of concomitant abdominal and subarachnoid hemorrhage due to SAM. A cranial CT examination of a 30-year-old male patient with a massive SAH (**a**). An abdominal CT examination of this patient revealed a retroperitoneal hemorrhage due to a rupture of the splenic artery. *Arrows* pointing to perihepatic and perisplenic blood, no active bleeding was observed in the initial CT scan (**b**). The DSA examination of the cervical and intracranial arteries showed remnants of previous dissections on both internal carotid arteries (**c**,**d**) and a blister aneurysm of the basilar artery (**e**). A cranial CT examination 13 days after the DSA examination confirmed the suspected recurrent SAH (**f**) and revealed a saccular aneurysm of the basilar trunk (**g**). A DSA examination 2 days later showed a large dissecting aneurysm of the basilar trunk (**h**), which was partially occluded with coils and covered with a p64 flow diverter (**i**). Follow-up DSA examination 11 months after the clinical onset (**j**): the dissecting aneurysm of the basilar artery is completely occluded following partial coil occlusion and flow diverter coverage

This 30-year-old, previously healthy male patient collapsed during his office work after complaining of severe headache, became hemodynamically unstable and was intubated and brought to the emergency room. There was no history of trauma. A computed tomographic (CT) examination of his body showed a massive retroperitoneal and subarachnoid hemorrhage (SAH) (Hunt and Hess IV, Fisher III) (Fig. 1a, b). The laparotomy showed a rupture of the splenic artery, hepatic and splenic lacerations and fragile abdominal vessels. He underwent emergent splenectomy and external ventricular shunting. Digital subtraction angiography (DSA) of the cervical and intracranial vessels 3 days after the initial event showed remnants of previous dissections of both internal carotid arteries (ICAs, Fig. 1c, d). On the middle section of the basilar artery (BA) a small blister aneurysm was recognized (Fig. 1e). Only 13 days after this first DSA examination a second SAH occurred (Fig. 1f) and was due to a large saccular aneurysm of the basilar trunk (Fig. 1g). The second DSA examination now showed a large dissecting aneurysm, which had developed from the previous blister aneurysm of the basilar artery (Fig. 1h). This aneurysm was partially occluded with coils and covered by a flow diverter (Fig. 1i). For this procedure the patient received 500 mg acetylsalicylic acid (ASA) intravenous (IV) and 180 mg ticagrelor per os (PO) together with a body weight adapted bolus of eptifibatide IV. The aneurysm was treated with coiling (2 × Deltamaxx, Codman) and flow diverter (FD) implantation (1 × p64, phenox). Complete coverage of the dissected segment of the basilar artery, including the orifice of the aneurysm was achieved. This procedure was well tolerated.

Based on the results of Multiplate and VerifyNow response tests, 1 × 500 mg ASA and 2 × 180 mg ticagrelor, both PO daily, were required to maintain sufficient platelet function inhibition due to thrombocytosis after splenectomy.

The patient was kept on dual antiplatelet therapy with ASA and ticagrelor for one year. The dosage was reduced stepwise during the course of the year while maintaining sufficient platelet function inhibition, monitored by repeated Multiplate and VerifyNow response tests to 1 × 100 mg ASA and 2 × 90 mg ticagrelor, both PO daily. Furthermore, the patient was treated with low molecular weight heparin for 6 weeks after the treatment, dexamethasone and etoricoxib for 6 weeks.

The course was further dominated by various issues like small bowel perforation, frontal subdural hematoma following ventricular shunting, revision laparotomies etc.

The patient recovered with a Barthel index of 90 five months after the clinical onset despite the fulminant beginning and course of his disease and a variety of subsequent abdominal complications. DSA of the cervical and cranial vasculature 11 months after the clinical onset confirmed the complete obliteration of the dissecting basilar artery aneurysm, with an unchanged appearance of the remaining vessels (Fig. 1j).

**Fig. 2:**
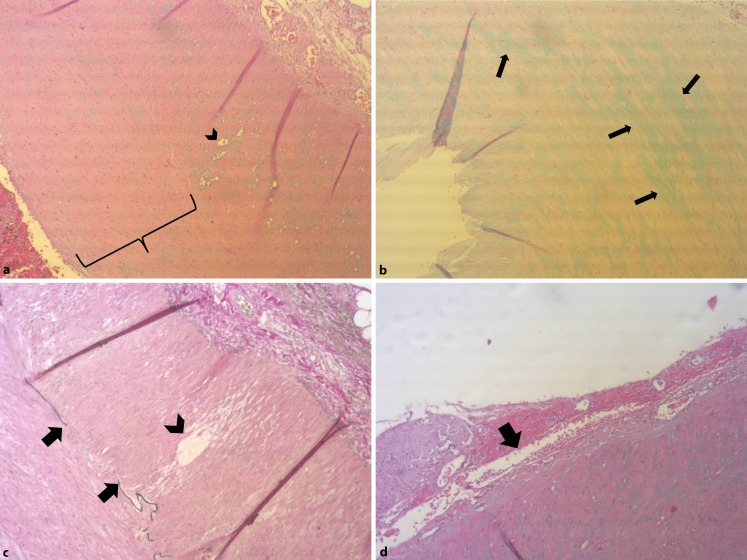
Histology of the resection specimen of the splenic artery: **a** Cross section of splenic artery wall: massive thickening of the intima (*bracket*) and cystic degeneration of the smooth media muscles of the media (*arrowhead*) (hematoxylin & eosin, × 100). **b** Mucoid degeneration of the splenic artery wall showing acid mucopolysaccharide depositions (*arrows*) in the media by Alcian blue positive stain (× 100). **c** *Arrows* showing large gaps in the internal elastic lamina demonstrating the fragmentation of the internal elastic lamina, *arrowhead*: vacuolization of the smoot media muscles of the media (Elastica van Gieson stain, × 100). **d** Focal submedial separation and hemorrhage between the adventitia and media (*arrow*) is also observed (hematoxylin & eosin, × 100)

The histologic specimen of the splenic artery showed an atypical architecture with loss of mediocytes, cystic degeneration, mucoid degeneration of lamina media, frequent rupture of internal elastic lamina, submedial bleeding and focal dissection (Fig. 2).

The presumptive diagnoses of the underlying vascular disorder include vascular Ehlers–Danlos syndrome, Loeys–Dietz syndrome, cystic medial necrosis Erdheim–Gsell and—possibly—segmental arterial mediolysis. The genetic examination revealed a heterozygotic mutation of the COL3A1 gene, which is not described so far but most likely pathogenic.

## Discussion

The imaging correlates in SAM reported in the literature include single or multiple dissection(s), intramural hematoma, arterial stenosis and occlusion, fusiform or saccular aneurysms. Splenic, coeliac, mesenteric and renal arteries are responsible for the abdominal manifestations. SAM has been described for the ICA, anterior and middle cerebral artery (ACA, MCA), vertebral artery (VA) and BA as well as for a spinal artery [5, 6].

The histopathological findings in SAM include patchy vacuolar degeneration of smooth muscle cells of the arterial tunica media, fibrin deposition at the media–adventitia junction, and mucoid material. The tunica media can be missing, bringing intima and adventitia in direct contact [7]. Alterations related to vasculitis or atherosclerosis are missing. The relation of SAM to fibromuscular dysplasia (FMD), cystic medial necrosis (CMN) and the vascular Ehlers–Danlos syndrome is a matter of debate. Leu [5] reports the histological findings in five patients with SAM of cervical and intracranial arteries. The focally distributed alterations of the media muscularis consisted of small necrotic areas, deposits of Alcian blue-positive substances and small cysts. The author emphasizes the similarities with the Erdheim–Gsell medionecrosis of the aorta. Yamada et al. [8] diagnosed both CMN and SAM in one patient and suspected a close relationship between these two disorders. Pickup et al. [4] described an association between SAM, mutations in the gene encoding type 3 procollagen (COL3A1) and the vascular Ehlers–Danlos syndrome. In their second case, features of cystic medial degeneration of the aorta were found.

The presumptive diagnoses of the underlying vascular disorder in our patient include vascular Ehlers–Danlos syndrome, Loeys–Dietz syndrome, cystic medial necrosis Erdheim–Gsell and—possibly—segmental arterial mediolysis. These diseases are known to show overlapping features [9]. The genetic examination revealed a heterozygotic mutation of the COL3A1 gene, which is known to be associated with type IV (vascular type) of the Ehlers–Danlos syndrome.

Inflammatory vasculopathies such as polyarteritis nodosa were excluded from the diagnosis as no inflammation of the vessel walls was histopathologically observed [7].

All in all, the vascular changes with cystic and mucoid degeneration of lamina media, rupture of internal elastic lamina, submedial bleeding and focal dissection associated with a COL3A1 mutation and the clinical and radiologic manifestations in our patient are typical for SAM, keeping in mind that an overlap with vascular Ehlers–Danlos syndrome is possible [10, 11].

The concomitant manifestation of SAM on abdominal and neurovascular arteries is rare. We identified 12 published cases [6, 12–22]. The key features of these reported cases are summarized in Table 1.

**Table 1:** Segmental arterial mediolysis (*SAM*) with concomitant visceral or thoracic and neurovascular manifestation. A review of 12 previously published cases

Authors	Patient age, gender	Visceral manifestation	Neurovascular manifestation	Histology	Clinical manifestation	Treatment
Kubo et al. 1992 [12]	56 female	Hepatic artery, ruptured aneurysm; splenic artery several incidental aneurysms	Right cervical ICA, ruptured aneurysm; left VA, incidental fusiform aneurysm	_	Cervical hematoma → abdominal hemorrhage	Surgery
Fuse et al. 1996 [13]	56 female	Gastroepiploic artery, ruptured aneurysm; gastric arteries, incidental	Left intradural ICA, ruptured aneurysm; right MCA bifurcation aneurysm, incidental	_	SAH → abdominal hemorrhage	Surgery
Sakata et al. 2002 [14]	48 male	Superior mesenteric artery, bilateral renal artery, left external iliac artery, dissections	Right VA and left ICA, fusiform dilatation, ruptured aneurysm	+	SAH	Conservative
Obara et al. 2006 [15]	52 male	Hepatic*, celiac*, superior mesenteric artery aneurysms and stenoses	Left ICA dissecting aneurysm*, stroke	+	Stroke	Surgery*
Ro et al. 2010 [16]	70 male	Right gastroepiploic artery, dissection, ruptured aneurysm; left gastric artery, dissection	Right VA, dissection, asymptomatic	+	Abdominal hemorrhage	Conservative
Stetler et al. 2012 [17]	59 female	Right hepatic artery, ruptured aneurysm*	Right ICA/PcomA, ruptured aneurysm*	_	SAH → abdominal hemorrhage	Coil occlusion*
Matsuda et al. 2012 [18]	58 male	Splenic, gastroepiploic, gastroduodenal, both renal artery aneurysms	Right ACA (A1* and distal), left VA, ruptured aneurysm	_	SAH	Surgery*
Alturkustani et al. 2013 [19]	47 male	Aortic dissection, incidental	Left VA (V4), ruptured fusiform aneurysm	+	SAH	Conservative
Cooke et al. 2013 [20]	45–55 male	Right internal mammary, celiac, both renal artery dissecting aneurysms	Left VA*, ruptured aneurysm	_	SAH	Coil occlusion*
Pillai et al. 2014 [21]	?	Celiac artery, dissection	Both ICAs, stroke	?	Stroke	?
Shinoda et al. 2016 [22]	47 male	Middle colic artery, ruptured fusiform aneurysm*	Extracranial VA, thyreocervical artery, incidental dissections; intradural VA, ruptured dissection*	+	SAH → abdominal hemorrhage	Coil occlusion*
Welch et al. 2017 [6]	61 male	Splenic artery aneurysm, hemorrhage	Posterior spinal artery aneurysm	_	Spinal SAH abdominal hemorrhage	Embolization

*ACA* anterior cerebral artery, *ICA* internal carotid artery, *PcomA* posterior communicating artery, *MCA* middle cerebral artery, *SAH* subarachnoidal hemorrhage, *VA* vertebral artery

There is no general treatment strategy for SAM-associated ruptured aneurysms. For abdominal aneurysms, endovascular treatment or surgery can be considered [23]. Intracranial dissecting aneurysms are usually not ideal surgical targets [24]. For vertebral artery dissections, parent vessel occlusion with coils is widely used [25]. For dissected intracranial arteries, which could not be occluded, stent reconstruction (with or without coil insertion) was for many years the only treatment option [26]. In the majority of cases, self-expanding stents developed to assist coil occlusion of aneurysms had been used. The implantation of flow diverters for this purpose has several advantages. The coverage of the dissected vessel is denser and the radial force is applied more evenly than with self-expanding stents. This may improve the re-adaptation of the separated vessel wall layers. There is very little hemodynamic impact of a self-expanding stent on a covered aneurysm. If, as in our patient, a dissection is the origin of a large saccular pseudoaneurysm, the hemodynamic effect of a flow diverter is advantageous to prevent (re-)rupture. Meanwhile flow diversion has become a recognized treatment option for intracranial dissections [27]. For this indication as for many others the required dual platelet function inhibition is a major drawback.

The initial presentation of SAM can be fulminant, as demonstrated by our patient. If this phase is survived, long-term disease-free survival has been reported [7].

## Conclusion

Structural disorders to the arterial tunica media may cause unusual clinical situations. Among those the concomitant abdominal and subarachnoid hemorrhage is a therapeutic challenge. In patients with unusual clinical presentations such as concomitant abdominal and subarachnoid hemorrhage it is important to keep structural vessel disorders such as SAM as a differential diagnosis in mind. Dissecting intracranial aneurysms are a good indication for flow diverter treatment. As the coincidence of abdominal and intracranial aneurysms is a rare event, genetic testing for the management of the patients and risk assessment is recommended.
